# Illicit drug use among students of a university in Southern Brazil

**DOI:** 10.11606/S1518-8787.2020054002176

**Published:** 2020-05-28

**Authors:** Gbènankpon Mathias Houvèssou, Isabel Oliveira Bierhals, Betina Daniele Flesch, Mariângela Freitas da Silveira

**Affiliations:** I Universidade Federal de Pelotas Faculdade de Medicina Programa de Pós-Graduação em Epidemiologia PelotasRS Brasil Universidade Federal de Pelotas. Faculdade de Medicina. Programa de Pós-Graduação em Epidemiologia. Pelotas, RS, Brasil

**Keywords:** Young Adult, Universities, Street Drugs, Drug-Seeking Behavior, Risk Factors

## Abstract

**OBJECTIVE:**

To describe drug consumption and the co-occurrence use of more than one illegal drug as well as associated factors in freshmen at a public university in Southern Brazil.

**METHODS:**

Cross-sectional study with census of students entering undergraduate courses in 2017. A total of 1,788 university students answered questions about illicit drug use. For analysis, ordinal logistic regression was used.

**RESULTS:**

Marijuana was the most consumed drug (lifetime: 42.1%; 30-day use: 22.7%), followed by hallucinogens (lifetime: 13.1%, 30-day use: 2.8%). Rates for lifetime use of 0, 1 and 2 or more drugs were 56.2%, 23.3% and 20.4%, respectively, and were associated with men (OR = 2.2; 95%CI:1.4–3.5), being at least 23 years old (OR = 2.7; 95%CI: 1.4–5.1), under 18 years old first experimentation with drugs (OR = 2.3; 95%CI: 1.3–3.9) and living with friends (OR = 2.0; 95%CI: 1.2–3.4). Rates for 30-day use of 0, 1 and 2 or more drugs were 76.8%; 18.1% and 5.1%, respectively, and were associated with being single, separated or widowed (OR = 3.2; 95%CI: 1.4–7.0), lower socioeconomic classes (OR = 0.3; 95%CI: 0.1–1.1; p = 0.001), under 18 years old first experimentation with drugs (OR = 1.8; 95%CI: 1.1–2.9) and living with friends (OR = 1.8 95%CI: 1.2–2.8).

**CONCLUSION:**

Results indicate that students are at greater risk of illicit drug-related health problems. Thus, a better understanding of this consumption should be pursued, as well as the development of a prevention plan.

## INTRODUCTION

It is estimated that 275 million people worldwide, approximately 5.6% of the world’s population aged between 15 and 64, used drugs at least once in 2016. Between 2000 and 2015, deaths due to drug use increased over 60%, of which approximately 168,000 were directly associated with drug-induced disorders (mainly overdoses). Furthermore, about 31 million people who used drugs suffered from use-related disorders^1^.

The university period is characterized by independence and distancing from parental supervision. In addition, it also features a transition period, including different living conditions from those of childhood and adolescence, new experiences, new bonds of friendship and family pressure^2^. Experimentation with illicit drug use at this stage is evident worldwide^2^, and the use of these substances within this period may increase^6^. Thus, undesirable results are a public health concern^7^, including unsafe sex^3^, depression^8^, poor academic performance^5^ and dropouts^8^.

In the United States, a national survey monitored the prevalence of drug use among students for 30 years, from eighth grade to adulthood, especially during the university phase^9^. Findings showed that 18.9% of university students admitted to using an illicit drug in the last 30 days, exceeding the 8% prevalence for the general population aged between 12 to 65^9,10^. In Brazil, according to the 2009 “First national survey on the use of alcohol, tobacco and other drugs among university students from 27 Brazilian capitals”, almost half of university students (48.7%) reported having used some illicit drug at least once in their lives, of which approximately one third (35.8%) had done so in the last 12 months and a quarter (25.9%) in the last 30 days. The most commonly used drugs within the 12 months before the survey were: marijuana (13.8%), amphetamines (10.5%), tranquilizers (8.4%), inhalants (6.5%) and hallucinogens (5%). Similarly, most frequently consumed drugs within the 30 days before the survey were: marijuana (9.1%), amphetamines (8.7%), tranquillizers (5.8%), inhalants (2.9%) and hallucinogens (2.8%)^11^.

Numerous social-, demographic-, economic- and family-related factors in this population are associated with substance use. Among many coexisting risk factors, there were: being male^12^, having a higher family income^16^, presenting other lifestyle characteristics and risk behaviors such as smoking and alcohol consumption^3^, and living with friends or people who use drugs^14^. On the other hand, protective factors included religious practice and living with relatives^3,4^, family communication^17^and commitment to education^18^.

Due to the potential damage on physical and mental health, acknowledging university students simultaneous consumption of more than one illicit drug has become increasingly important^19^. Although many studies have evaluated illicit drug use in this population^14,15,20^, the simultaneous use of more than one substance has not been thorough studied. Thus, our study aimed to describe drug consumption among university students and evaluate the co-occurrence consumption of more than one illicit drug, including associated factors.

## METHODS

Using a census format, this cross-sectional university-based study was conducted with undergraduate students enrolled at the *Universidade Federal de Pelotas* (UFPel) in 2017 to determine this population’s health characteristics. This was a census study conducted by the UFPel Graduate Epidemiology Program and is part of the University Student Health – UFPel (SEU) research consortium. UFPel is located in the south of Rio Grande do Sul and was created in 1969. It has 96 presential courses, receiving about 3,000 new students each semester. In 2018, the University had 16,461 undergraduate students (http: // portal. ufpel.edu.br/historico).

A previously tested digital version of the questionnaire was developed in the REDCap (*Research Electronic Data Capture*) system^24^. The questionnaire consisted of two blocks: general and specific. The general block included questions regarding the subject’s general data such as sex, age, major, socioeconomic level, marital status, etc. The specific block included questions of interest, such as drug consumption. The questionnaire was anonymous and self-applied using tablets.

Data were collected from November 2017 to May 2018. To facilitate student recruitment, the University provided information to all 2017 first-semester students. Course coordinators and professors were contacted to reserve time so students could answer the questionnaire, which took approximately 40 minutes. Teams of master’s students were allocated in each classroom to answer the students’ questions.

All students enrolled in UFPel degree programs for the first semester of 2017, either in their second or third semesters at the time of the interview, and aged 18 years or older were considered eligible for the study. So, as the study consisted of a census, every student who entered any degree program in 2017 and was still attending the course during data collection was invited to answer the survey. Those enrolled in distance learning programs or who had a visual or hearing impairment or any other condition preventing them from responding to the questionnaire were considered ineligible.

Dependent variables included use of cocaine, solvents and inhalants (*loló*/*lança-perfume* [chloroform and ether base], rubber cement, paint thinner, benzene, nail polish, gasoline), ecstasy (MDMA), hallucinogens (LSD, mushroom or lily tea) and marijuana. The questionnaire was based on the *II* Second National Survey on Drugs and Alcohol^25^, whose questions evaluated both lifetime and 30 days prior consumption categorized into the following rates: 0 = no use, 1 = used one drug, 2+ = used two or more drugs.

Independent variables were: sex (female, male); age (18–19, 20–22, and ≥ 23 years), used to avoid categories with very few individuals; skin color (white, black/brown/other); marital status (married or single/separated/widowed); religious practice (yes, no); living situation (with family/partner, alone, with friends); socioeconomic level (based on Brazilian Association of Research Companies [Abep] criteria: classes A, B, C, D and E)^26^; major depression (based on the Patient Health Questionnaire-9, with a cutoff point of ≥ 9);^27^ stressful events (including giving up or postponing important moments of leisure, having financial problems or feeling worried, anxious, discouraged, or tense because of the academic activities; feeling alone or lacking support; suffering discrimination from peers or professors; being pressured to perform well; being verbally or physically assaulted by peers; experiencing conflict with professor(s); undergoing changes in living habits; being disappointed with teaching quality), scored on the following scale: happened, but did not affect me; happened, but I was little affected; happened, and I was partially affected; happened and deeply affected me; did not happen to me. The last variable was dichotomized later, with stressor events coded as “yes” only in cases in which participants reported having been deeply affected by at least one event; area of major (exact sciences and soil sciences/agronomy, health and biological sciences, applied social sciences and humanities, linguistics, arts and languages), morning or evening classes, and age of first experience with alcohol, tobacco or drugs (< 18 or ≥ 18 years).

Statistical analyses were performed using Stata 12.1® (Stata Corp., College Station, Texas, USA). Initially, a descriptive analysis of independent variables and outcomes was performed. For associations between them, ordinal logistic regression was used to provide the crude and adjusted odds ratios and their respective 95% confidence intervals (95%CI). Possible confounding factors were analyzed following a conceptual model, and variables considered relevant were included in the bivariate analysis.

The study was approved by the UFPel Ethics Committee of Faculdade de Medicina (Protocol No. 79250317.0.0000.5317). The study was explained, including assurances of secrecy and confidentiality, to all participants, who signed an informed consent form.

## RESULTS

A total of 2,706 students were considered eligible for the survey. After successive contact attempts, there were 792 losses and 49 denials, comprising 31.1% of the total sample. Among these, 52.8% were men, 46.7% were 23 years old or older, and 38.3% were exact sciences and soil sciences/agronomy majors. The final sample comprised 1,865 students, of which 77 did not present complete information on illicit drug issues and were excluded from the analyses. There were 1,788 participants remaining, of whom 54.6% were women, and 72.2% reported being white (Table 1). Most of them were among the 18-22 age range (74.1%), with a mean age of 22.2 years (± 6.6 years). A total of 91.4% of the participants were either single, separated or widowed, and 59.2% were from the two highest social classes (A and B). Approximately 68.2% reported no religious practice, 61.8% lived with family members or partners, 55.5% presented depression symptoms, and 14.7% had undergone a stressful event. Regarding major, 34.5% were enrolled in applied social sciences and humanities courses, and the majority (53.6%) studied during the morning.

**Table 1:** Demographic, socioeconomic, behavioral, and academic profile of university students aged 18 years or older. Pelotas, Brazil, 2018 (n = 1,788).

Variable	n	%
Sex		
Female	975	54.6
Male	811	45.4
Age (years)		
18–19	738	41.6
20–22	578	32.5
23 or older	460	25.9
Self-reported skin color		
White	1289	72.2
Black/Brown/Other	497	27.8
Marital status		
Married or in consensual union	153	8.6
Single/Separated/Divorced/Widowed	1635	91.4
Socioeconomic class (Abep criterion)		
A	254	14.8
B	759	44.4
C	623	36.4
D/E	75	4.4
Religion		
No	1221	68.3
Yes	566	31.7
Depressive symptoms (PHQ9 ≥9)		
No	796	44.5
Yes	992	55.5
Exposure to stressful event		
No	1525	85.3
Yes	263	14.7
Academic major		
Exact sciences and soil/agricultural sciences	523	29.3
Health and life sciences	312	17.4
Applied social sciences and humanities	617	34.5
Linguistics/arts and letters	336	18.8
Class time		
Morning	959	53.6
Evening	829	46.4
Living arrangement		
With family or spouse/companion	1104	61.8
Alone	222	12.4
With friends	460	25.8
Age at first alcohol intake		
< 18	1463	81.8
≥ 18	325	18.2
Age at first tobacco use		
< 18	305	64.2
≥ 18	170	35.8
Age at first illicit drug use		
< 18	398	52.0
≥ 18	368	48.0

Figure shows the prevalence of lifetime and 30-day drug use and consumption rates. Lifetime rates for 0, 1 and 2 or more drugs were 56.2%, 23.3% and 20.4%, respectively; 30-day use rates for 0, 1 and 2 or more drugs were 76.8%, 18.1% and 5.1%, respectively. Marijuana was the most frequently used drug, with lifetime and 30-day use rates of 42.1% and 22.7%, respectively, followed by hallucinogens (lifetime: 13.1%; 30-day use: 2.8%). The least common drugs were solvents and inhalants (lifetime use: 9.9%; 30-day use: 1.5%) and cocaine (lifetime: 9.9%; 30-day use: 1.8%) (Figure).

**Figure:**
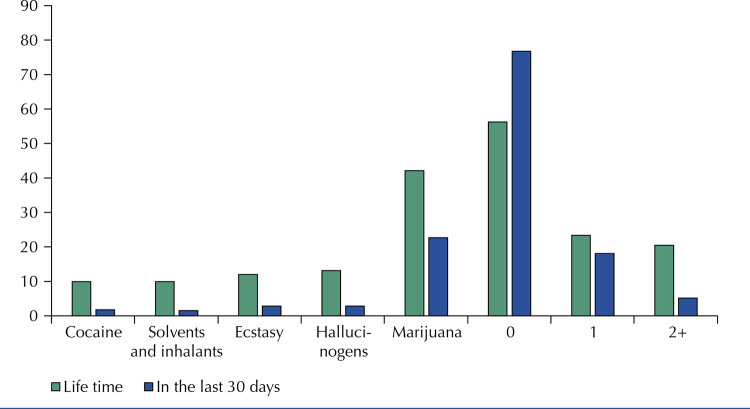
Prevalence of drug use among university students aged 18 or older in lifetime and 30 days before the survey, Pelotas, RS, 2018.

Table 2 describes factors associated with lifetime drug use rates. After adjustment for possible confounding factors, men were 2.2 times more likely (95%CI: 1.4–3.5) to be in a higher drug consumption category than women (p = 0.001), and individuals aged 23 years or older were 2.7 times more likely (95%CI: 1.4–5.1) to be in a higher consumption category than 18 to 19-year-olds (p = 0.003). Those who first experimented with drugs before the age of 18 were 2.3 times more likely (95%CI: 1.3–3.9) to be in a higher consumption category than those who first experimented with drugs at 18 or older (p = 0.003). Those who reported experimenting drugs with friends were twice as likely (95%CI: 1.2–3.4) to be in a higher drug consumption category than those who lived with relatives or partners (p = 0.020).

**Table 2:** Factors associated with lifetime drugs use rate (0, 1, 2+) among university students aged 18 or older.

Level	Variable	Crude analysis			Adjusted analysis		
	
		OR	95%CI	p-value	OR	95%CI	p-value
1	Sex			< 0.001			0.001
	Female	1			1		
	Male	1.5	1.2–1.8		2.2	1.4–3.5	
1	Age (years)			0.003			0.003*
	18–19	1			1		
	20–22	1.4	1.1–1.7		1.3	0.7–2.2	
	23 or older	1.3	1.1–1.7		2.7	1.4–5.1	
1	Self-reported skin color			0.141			0.061
	Black/Brown/Other	1			1		
	White	1.2	0.9–1.4		1.2	0.9–1.4	
2	Marital status			0.294			-
	Married or in consensual union	1			-		
	Single/Separated/Divorced/Widowed	1.2	0.9–1.7		-	- -	
2	Socioeconomic class (Abep criterion)			0.078			0.077
	A	1			1		
	B	0.8	0.6–1.0		0.8	0.6–1.0	
	C	0.7	0.6–10		0.7	0.5–1.0	
	D/E	0.6	0.3–0.9		0.6	0.3–0.9	
3	Religion			< 0.001			0.446
	No	2.1	1.7–2.5		1.2	0.7–2.2	
	Yes	1			1		
3	Depressive symptoms (PHQ9 ≥9)			< 0.001			0.757
	No	1			1		
	Yes	1.7	1.4–2.0		0.9	0.6–1.5	
3	Exposure to stressful eventz			0.722			-
	No	1			-		
	Yes	1.0	0.8–1.3		-	-	
3	Age at first alcohol intake			< 0.001			0.506
	< 18	3.6	2.7–4.7		1.4	0.6–3.3	
	≥ 18	1			1		
3	Age at first tobacco use			< 0.001			0.050
	< 18	2.3	1.6–3.2		1.7	0.9–2.9	
	≥ 18	1			1		
3	Age at first illicit drug use			< 0.001			0.003
	< 18	2.7	2.0–3.7		2.3	1.3–3.9	
	≥ 18	1			1		
3	Academic major			< 0.001			0.329
	Exact sciences and soil/agricultural sciences	1			1		
	Health and life sciences	1.3	1.0–1.8		2.0	0.9–4.2	
	Applied social sciences and humanities	1.3	1.0–1.6		1.3	0.7–2.7	
	Linguistics/arts and letters	1.7	1.3–2.3		1.4	0.8–3.1	
4	Class time			0.012			0.480
	Morning	1			1		
	Evening	1.3	1.0–1.5		1.2	0.7–1.9	
4	Living arrangement			< 0.001			0.020
	With family or spouse/companion	1			1		
	Alone	1.4	1.1–1.9		2.0	0.9–3.9	
	With friends	2.6	2.1–3.2		2.0	1.2–3.4	

Level 1: adjusted for sex, age, self-reported skin color; Level 2: previous adjustment + socioeconomic class; Level 3: previous adjustment + religion, depressive symptoms, age at first alcohol intake, age at first tobacco use, age at first illicit drugs use and academic major; Level 4: previous adjustment + class time and living arrangement.

* p-value of linear tendency

Table 3 shows factors associated with 30-day drug use rates. Individuals who were single, separated or widowed were 3.2 times more likely (95%CI: 1.4–7.0) to be in a higher consumption category than those married or in stable union (p = 0.005). Regarding socioeconomic level, those in class B and C had 0.5 (95%CI: 0.3–0.8) and 0.3 (95%CI: 0.2–0.5) times less chance, respectively, than those in class A (p = 0.001) to be in a higher consumption category. Those who experimented with drugs before the age of 18 were 1.8 times more likely (95%CI: 1.1–2.9) to be in a higher drug consumption category than those who did so at 18 or older (p = 0.019), as were those who reported living with friends (95%CI: 1.2–2.8) compared with those living with relatives or partners (p = 0.026).

**Table 3:** Factors associated with the 30 days before drugs use rate (0, 1, 2+) among university students aged 18 or older.

Level	Variable	Crude analysis			Adjusted analysis		
	
		OR	95%CI	p-value	OR	95%CI	p-value
1	Sex			0.039			0.559
	Female	1			1		
	Male	1.3	1.0–1.6		1.1	0.7–1.7	
1	Age (years)			0.250			-
	18–19	1			-		
	20–22	1.0	0.8–1.3		-	-	
	23 or older	0.8	0.6–1.1		-	-	
1	Self-reported skin color			0.506			-
	Black/Brown/Other	1			-		
	White	1.1	0.9–1.4		-	-	
2	Marital status			< 0.001			0.005
	Married or in consensual union	1			1		
	Single/Separated/Divorced/Widowed	2.2	1.3–3.5		3.2	1.4–7.0	
2	Socioeconomic class (Abep criterion)			0.040			0.001
	A	1			1		
	B	0.9	0.6–1.2		0.5	0.3–0.8	
	C	0.7	0.5–0.9		0.3	0.2–0.5	
	D/E	0.6	0.3–1.2		0.3	0.1–1.1	
3	Religion			< 0.001			0.874
	No	1.9	1.5–2.9		0.9	0.6–1.5	
	Yes	1			1		
3	Depressive symptoms (PHQ9 ≥9)			< 0.001			0.411
	No	1			1		
	Yes	1.7	1.3–2.1		1.2	0.8–1.9	
3	Exposure to stressful event			0.166			0.732
	No	1			1		
	Yes	1.2	0.9–1.7		1.1	0.6–1.8	
3	Age at first alcohol intake			< 0.001			0.109
	< 18	3.4	2.3–5.0		1.9	0.9–4.3	
	≥ 18	1			1		
3	Age at first tobacco use			0.117			0.094
	< 18	1.3	0.9–1.9		0.7	0.4–1.1	
	≥ 18	1			1		
3	Age at first illicit drug use			< 0.001			0.019
	< 18	1.8	1.3–2.3		1.8	1.1–2.9	
	≥ 18	1			1		
3	Academic major			0.005			0.557
	Exact sciences and soil/agricultural sciences	1			1		
	Health and life sciences	1.1	0.8–1.5		1.6	0.8–3.1	
	Applied social sciences and humanities	1.2	0.9–1.6		1.5	0.8–2.8	
	Linguistics/arts and letters	1.7	1.3–2.4		1.3	0.7–2.5	
4	Class time			0.573			-
	Morning	1			-		
	Evening	1.1	0.9–1.3		-	-	
4	Living arrangement			< 0.001			0.026
	With family or spouse/companion	1			1		
	Alone	1.9	1.4–2.7		1.7	0.9–3.2	
	With friends	3.2	2.5–4.1		1.8	1.2–2.8	

Level 1: adjusted for sex, socioeconomic class, marital status; level 2: previous adjustment + religion, depressive symptoms, stressful event, age at first alcohol intake, age at first tobacco use, age at first illicit drug use and academic major; level 3: previous adjustment + living arrangement.

## DISCUSSION

This study described the consumption of illicit drugs among university students and assessed consumption rates, as well as their associated factors. Marijuana was the illicit drug most commonly consumed by students, followed by hallucinogens. Regarding associated factors, being male, 23 years old or older, having experimented with drugs before the age of 18 and living alone or with friends were risk factors for lifetime drug use; being single, separated or widowed, having higher income, and having experimented with drugs before the age of 18 were risk factors for consumption in the 30 days prior to the interview.

Rates used in this study described the prevalence of having used no drugs, at least one drug and two or more drugs. Pilatti et al.^20^ (2013) found a 33.3% lifetime prevalence for using at least one drug (marijuana, cocaine, inhalant or ecstasy) and a 17.4% 30-day prevalence. Although 30-day use prevalence were similar to that found in this study, our lifetime use prevalence was higher.^20^ As a higher prevalence could be expected, considering the study population was first to fifth year university students,^20^ one possible justification would be that Pilatti et al.^20^ (2013) did not evaluate hallucinogens, the second most commonly used drug in this study. Andrade et al.^12^ (2012) interviewed university students nationwide from all years and found 48.7% lifetime prevalence for the use of one drug and 25.9% for 30-day use, slightly higher than those observed in our study. This could be due to the fact that these authors included more drug varieties than this study did (as amphetamines, anticholinergics, tranquilizers, opioid analgesics, barbiturates, anabolic androgenic steroids), as well as that students were evaluated in later university years.

Although many studies have reported marijuana as the most commonly used illicit drug by university students^14,16,20,22^, others reported inhalants and solvents to be the most common^13,28^. A nationwide Brazilian study found a lower prevalence of marijuana use (26.1% lifetime; 9.1% 30-day use), as well as lower lifetime prevalence for all other drugs except inhalants, whose prevalence was substantially higher (20.4%) than that of this study^11^. Canuto et al.^13^ (2006) analyzed first-year Brazilian university students, finding greater lifetime inhalant consumption (23.0%)^13^. The highest prevalence for this substance was associated with bars/nightclubs consumption and obtaining the substance with friends, suggesting that its use occurs in a recreational context^13^.

In our study, the age of first drug experimentation and the student’s living situation were associated with both outcomes (lifetime and 30-day use rates). First experimentation before 18 years old increased the chance of being in a higher consumption category than first experimentation after 18 years old (p = 0.003 for lifetime and p = 0.019 for 30-day rates). Other studies have found similar results, indicating that the risk of abusing or developing an addiction to illicit drugs is greater for individuals who begin using it during adolescence rather than in adulthood^29,30^. For example, a 2012 United States study with adults being treated for drug dependence found that of those who first experimented with marijuana at 14 years old or younger, 13.2% were classified as addicted, six times higher than the rate of adults who first experimented with it at 18 years old or older^31^.

Regarding living situation, individuals living alone or with friends were more likely to be in a higher drug consumption category than those living with relatives or partners (p = 0.020 for lifetime and p = 0.026 for 30-day rates), corroborating the literature^14^. Living outside family care is reported as a facilitator of health risk behaviors. Individuals who leave their parents’ home to study and remain far from them, for instance, may have a greater sense of freedom towards new experiences and curiosities, such as the use of licit and illicit drugs^32^.

Regarding lifetime drug use rates, sex (p = 0.001) and age (p = 0.003) were also associated variables. Men were more likely to be in a higher drug consumption category than women. This finding could be explained by the fact that women perceive drugs as more dangerous^33^. It may also reflect a greater social tolerance for men who use drugs and a greater social stigma for women^33^. Biologically speaking, drug metabolism is different in men and women, with men generally having higher clearance rates^34^. A similar result was found by Silva et al.^16^ (2006) and McCabe et al.^35^ (2007), who assessed 12-month illicit drug use. Nevertheless, sex was not associated with 30-day use rates in this study adjusted analysis (p = 0.559).

In our study, age increase is proportional to the increase in chances of being in a higher drug consumption category, corroborating Passos et al.^23^ (2006), who evaluated young people, mostly between 17 and 23 years old and found that as age increases, so does illicit drug use^23^. Despite the trend found in this study and that of Passos^23^ et al., involving very similar age groups, nearly all included individuals were between 18 and 24 years old, age group of greatest drug consumption^36^. A study on young American adults between 18 and 29 years old found that this group has higher rates for drug use, abuse and dependence than older age groups^37^. The difference between the results of the present study and these of Johnston et. al^37^ could be due to the fact that after young adulthood some social responsibilities (adult roles) are assumed, such as having children, and the use of illicit drugs decreases^38^.

Marital status and socioeconomic level were associated with 30-day use rates. Higher illicit drug use was observed among single, separated and widowed individuals (p = 0.005). A longitudinal study by Duncan et al^39^. (2006) that assessed the effects of marriage and cohabitation on drug use, reported a decrease in the consumption of marijuana among married couples, presenting a protective effect against drug use^39^. Thus, it has been observed that socially deviant behavior, such as illicit drug use, are incompatible with traditional adult social roles, such as marriage^38^, which could justify the finding of the present study that single individuals consume more drugs.

Socioeconomic level was associated with 30-day drug use, in which individuals with higher incomes presented higher consumption (p = 0.001). Other studies found similar results^16,40^. This was also consistent with the findings of Humensky (2010)^40^, who evaluated the relationship between adolescent socioeconomic status and 30-day drug use in early adulthood. Among people whose parents had higher incomes, a higher use of marijuana and cocaine was found^40^, implying that illicit substances demand is price sensitive^41^, This finding can be firstly explained by the fact that it is easier for students with higher incomes to acquire drugs^42^ and, secondly because parents with higher incomes generally have a greater commitment to work, contributing less to the monitoring of their children’s behavior^43^. A study by Martins et. al^44^ (2008) supports this argument by indicating that parental monitoring or positive parenting practices reduce the risk of adolescent drug use and abuse^44^. Finally, students of lower economic classes tend to consider university education a path to social mobility^45^, whereas richer students celebrate youth by socializing and engaging in deviant behaviors^45^.

This study has some limitations. Due to the cross-sectional design, the main limitation of the study is a possible reverse causality bias in some associations, such as the marital status variable. Losses to follow-up and refusals were significant, restricting findings extrapolation, with a greater conflict among men, students aged 23 and older, and students of exact sciences and soil/agricultural science programs; which may have led to selection bias. Students not reached could have presented higher drug use, leading to difficulties to attend classes and a greater possibility of abandoning university studies. In addition, as losses were greater among men and students of exact sciences and soil/agricultural sciences, also those with a greater chance of using any substance, the prevalence found could be underestimated. Some students may have preferred not to report illicit drug use, even with guaranteed anonymity. Thus, drug use prevalence should be interpreted as minimum estimates of the actual values, which could be higher. However, using a self-administered confidential questionnaire is a standard procedure for obtaining information about this type of behavior. Another limitation is the generalization of the findings to the entire university population, since only new students were included in the study population. As it was performed in a consortium format, the number of questions in the study was also limited, preventing a more detailed outcome assessment, such as when drug experimentation first occurred and situations and places where drugs were consumed. The long duration of data collection is also one of the limitations of this study. Students who were interviewed at the end of fieldwork are more exposed to the university environment than those who were interviewed at the beginning.

The study has some strengths, as it contributes to the comprehension of the use of illegal drugs and its associated factors among university students.

In conclusion, marijuana was the most frequently illicit drug used by students, and 5.1% of the participants reported using two or more illicit drugs in the 30 days before the survey, representing the group at highest risk of dependence and possible psychological distress. Our results are expected to contribute to a better understanding of the context of substance use in this population.
